# A cross-laboratory preclinical study on the effectiveness of interleukin-1 receptor antagonist in stroke

**DOI:** 10.1177/0271678X15606714

**Published:** 2015-09-30

**Authors:** Samaneh Maysami, Raymond Wong, Jesus M Pradillo, Adam Denes, Hiramani Dhungana, Tarja Malm, Jari Koistinaho, Cyrille Orset, Mahbubur Rahman, Marina Rubio, Markus Schwaninger, Denis Vivien, Philip M Bath, Nancy J Rothwell, Stuart M Allan

**Affiliations:** 1 Faculty of Life Sciences, University of Manchester, Manchester, UK; 2 Department of Pharmacology, Medicine School, University Complutense of Madrid, Spain; 3 Laboratory of Neuroimmunology, Institute of Experimental Medicine, Budapest, Hungary; 4 Department of Neurobiology, A. I. Virtanen Institute for Molecular Sciences, University of Eastern Finland, Kuopio, Finland; 5 Inserm, Inserm UMR-S U919, Université de Caen Basse Normandie, GIP Cyceron, Caen, France; 6 Institute of Experimental and Clinical Pharmacology and Toxicology, University of Lübeck, Lübeck, Germany; 7 Department of Pharmaceutical Sciences, North South University, Bashundhara, Dhaka, Bangladesh; 8 Stroke, Division of Clinical Neuroscience, University of Nottingham, City Hospital Campus, Nottingham, UK

**Keywords:** Acute stroke, inflammation, animal models, neuroprotection, experimental

## Abstract

Stroke represents a global challenge and is a leading cause of permanent disability worldwide. Despite much effort, translation of research findings to clinical benefit has not yet been successful. Failure of neuroprotection trials is considered, in part, due to the low quality of preclinical studies, low level of reproducibility across different laboratories and that stroke co-morbidities have not been fully considered in experimental models. More rigorous testing of new drug candidates in different experimental models of stroke and initiation of preclinical cross-laboratory studies have been suggested as ways to improve translation. However, to our knowledge, no drugs currently in clinical stroke trials have been investigated in preclinical cross-laboratory studies. The cytokine interleukin 1 is a key mediator of neuronal injury, and the naturally occurring interleukin 1 receptor antagonist has been reported as beneficial in experimental studies of stroke. In the present paper, we report on a preclinical cross-laboratory stroke trial designed to investigate the efficacy of interleukin 1 receptor antagonist in different research laboratories across Europe. Our results strongly support the therapeutic potential of interleukin 1 receptor antagonist in experimental stroke and provide further evidence that interleukin 1 receptor antagonist should be evaluated in more extensive clinical stroke trials.

## Introduction

In the UK and Europe, the third leading cause of death after heart disease and cancers is stroke,^1^ which causes greater disability than any other condition.^2^ Translation of preclinical findings in stroke to clinical benefit has not been successful to date and treatment opportunities are limited to clot lysis and, as demonstrated recently, thrombectomy.^3^ However, though these interventions can provide benefit, they are restricted to a subset of stroke patients and more widely applicable treatments are still urgently required, including ways to minimise brain injury before reperfusion strategies can be implemented. Failure of clinical stroke trials is thought to be largely due to the poor quality of preclinical studies, low level of reproducibility across research labs and to the fact that common co-morbidities in stroke (e.g. age, atherosclerosis, obesity, etc.) have not been appropriately considered in preclinical modelling. The Stroke Therapy Academic Industry Roundtable (STAIR) has established detailed recommendations for the evaluation of preclinical and clinical acute stroke treatments, with specific guidelines on improving the quality of experimental studies.^4^ In addition, initiation of international, multicentre randomised preclinical trials in translational stroke research has been suggested,^5,6^ which would largely support drug development and selection of candidates for clinical testing. To our knowledge, no stroke treatment has yet been tested in a cross-laboratory preclinical study.

We have shown that inflammatory mechanisms are central to the pathophysiology of stroke.^7,8^ In particular, the cytokine interleukin 1 (IL-1) is a key player in the pro-inflammatory response that underpins the inflammatory component of stroke and other forms of brain injury.^9^ Attenuation of inflammation after brain injury by inhibiting endogenous IL-1 is an attractive therapeutic strategy and use of the naturally occurring IL-1 receptor antagonist (IL-1Ra) has been shown as protective in preclinical models of stroke,^10^ subarachnoid haemorrhage^11^ and traumatic brain injury.^12^ IL-1Ra has also shown promise in a randomised phase II trial in acute ischaemic stroke, which found it to be safe and well tolerated.^13^ Despite the strong evidence in support of IL-1Ra as a treatment for stroke, there are significant translational challenges in taking successful outcomes from the preclinical to clinical setting. Methodological quality and differences in experimental design between studies and laboratories can have a profound effect on outcome.^14^ Therefore, we initiated a preclinical cross-laboratory study to evaluate the therapeutic potential of IL-1Ra following middle cerebral artery occlusion (MCAo) in the mouse.

## Materials and methods

### Experimental design

Several preclinical stroke research groups across Europe were approached to participate in a cross-laboratory study to confirm efficacy of IL-1Ra in experimental stroke. Invited centres were asked to respond to the following questions: (1) which species (e.g. mouse, rat, other?) and strain do/could you use? (2) Which cerebral ischaemia model(s) are you set up to do? (3) Which short- and long-term outcome measures (including behaviour) do/could you use? (4) Could you do imaging (e.g. serial MRI)? (5) What clinically relevant/co-morbid animal models do/could you use? Following this initial invitation, four European centres (Finland, France, Germany, Hungary) were recruited to join the study in addition to the coordinating centre in Manchester. On the basis of the responses obtained regarding capabilities in each of these centres, a study was then designed to establish the effects of IL-1Ra in both permanent and transient models of MCAo with short (24 h), medium (7 d) and longer term (28 d) assessment of infarct (histology and/or MRI) and functional outcome. Based on their initial experiments, a standard operating procedure (SOP) was drawn up by the Manchester group, and distributed to all participating centres. However, due to local practices and availability of resources, the SOP could not be adhered to and individual research groups conducted the experiments largely according to normal practice, details of which are described below. All studies followed the ARRIVE guidelines.^15^ Summary details of each study are provided in Table 1.

**Table 1.** Characteristics of study.

Study	Species (strain)	Sex	Age (m)	Anesthetic	Model (occlusion time)	IL-1Ra (mg/kg)	Dose times (min)	Lesion and functional outcome	Lesion/ oedema measure	Lesion measured	Oedema measured	Lesion/oedema measured (day)	Neurologicalscore (day)	Functional outcome (day)
Budapest 2013	Mouse (C57 BL/6)	M	2.5	Isoflurane	Transient (Fil) 45 min	100 s.c.	30 + 180	Y	Hist Stain (cresyl)	y	y	7	1 + 7 (Bederson)	Corner 1 + 7
Kuopio 2011	Mouse (BALB/C)	M	10	Halothane	Permanent (Electrocoag)	100 s.c.	30 + 180	Y	MRI	y	n	3 + 28	none	Catwalk 3 + 3 + 7 + 14 + 21 + 28
M’chester 2012	Mouse (BALB/C)	M	2.5	Isoflurane	Permanent (Electrocoag)	100 s.c.	30 + 180	Y	MRI	y	y	1 + 7	1 + 2 + 7 (Hunter)	Corner 1 + 2 + 7
M’chester 2012	Mouse (BALB/C)	M	10	Isoflurane	Permanent (Electrocoag)	100 s.c.	30 + 180	Y	MRI	y	y	1 + 7	1 + 2 + 7 + 28 (Hunter)	Corner 1 + 2 + 7 + 28
M’chester 2010	Mouse (C57 BL/6)	M	2.5	Isoflurane	Transient (Fil) 30 min	100 s.c.	30 + 180	N	Hist Stain (cresyl)	y	n	7	none	none
M’chester 2011	Mouse (C57 BL/6)	M	2.5	Isoflurane	Transient (Fil/Fil + TPA) 30 min	100 s.c.	30 + 180	N	Hist Stain (cresyl)	y	y	7	0 + 1 (21 point)	none
Caen 2012	Mouse (C57 BL/6)	M	2.5	Isoflurane	Transient (Thrombin/ Thrombin + TPA) 30 min	100 s.c.	30 + 180	N	Hist Stain (cresyl)	y	n	7	none	none
Lübeck 2011	Mouse (C57 BL/6)	M	2.5	Tribro- methanol	Permanent (Electrocoag)	100 s.c.	30 + 180	Y	Hist Stain (silver)	y	y	7	none	Corner 1 + 2 + 7

#### Induction of ischaemia

All studies used male mice (three studies with BALB/C and three using C57BL/6) with four studies using transient models of occlusion (confirmed by blood-flow measurements by laser Doppler) and four using permanent models (confirmed visually). The length of occlusion times varied between studies for transient strokes with three studies giving 30 min of occlusion and another 45 min. Studies used animals of either 2.5 months (six studies) or 10 months (two studies) of age. Isoflurane was used as anaesthetic in six studies, halothane in one and the injectable tribromethanol in another. No analysis was undertaken of whether the different anaesthetics affected outcome as only one study for each.

#### Drug treatment

Placebo or IL-1Ra (100 mg/kg) was prepared in the lead lab (Manchester) and coded (A or B) by an independent researcher. Individual tubes labelled A and B were then sent to the different labs taking part in the study. Animals were randomly assigned either treatment A or B in the separate studies with blinded analysis of outcomes in individual studies being completed by each participating centre. Full datasets were received from each of these centres by the lead lab before the allocation code was broken and group identity revealed. All studies used subcutaneous (s.c.) injection of IL-1Ra or placebo at 30 and 180 min after MCAo.

#### Assessment of ischaemic injury

Lesion volumes and oedema were measured by histological staining (three studies) or MRI (three studies). All lesion volumes and oedema measured by staining methods were assessed seven days post-occlusion while MRI measurements ranged from 1 to 28 days post-stroke. All lesion and oedema values were reported in mm^3^.

#### Neurological scoring

Not all centres had equal capacity with regard to running behavioural assessments and therefore were provided flexibility to the tests used to determine neurological deficits post-stroke. Tests used for functional assessment of outcomes included the corner (four studies) and catwalk tests (one study). Detection of sensorimotor asymmetry by the corner test ranged from 1 to 28 days after MCAo and catwalk ranged from 3 days prior MCAo (base line) to 28 days after. Sensorimotor asymmetry (i.e. corner test) was measured using laterality index, i.e. ipsilateral turns-contralateral turns/total turns. The higher the score, the more deficits over a trial period. Neurological deficit scoring was recorded in four studies, all lasting up to seven days (four studies), while two studies went on longer to 28 days. Recordings of neurological deficit ranged from 0 to 28 days post-stroke. Two studies used a modified neurological deficit scoring system based on Hunter et al.,^16^ one study used Bederson et al.^17^ and another study assessed behaviour based on a modified version of Garcia et al.^18^ The majority of neurological scores accumulates points for increasing deficits. However, the neurological deficit scoring system based on Hunter is the opposite and more points are scored for the lack of deficits. In order to incorporate data from studies using the Hunter scoring system, Hunter scores were deducted from total maximum score (22 points) to produce an inverse value that can be incorporated into analysis, i.e. Hunter values showing high scores (lack of deficit) will result in low values representing a score for neurological deficits present.

#### Mortality

Only deaths post-treatment were analysed and any earlier culling (e.g. due to inadequate occlusion – as determined by using laser Doppler) does not amount to attrition bias and therefore is not included in the meta-analysis. However, all animals that were culled due to poor condition post-stroke (e.g. they exhibited barrel-rolling) were included in the analysis. Details of all deaths, whether included in the meta-analysis or not were recorded by the coordinating centre (see section below).

#### Randomisation/blinding

Animals were randomised to treatment according to local practice in participating centres as follows: Budapest – after establishment of group sizes, GraphPad Random Number Generator (randomisation for two groups) was used to assign numbers obtained to individual mice (blocked for cages, to avoid cage/litter effect); Caen – a randomisation table was established prior to the study, and animals were assigned on the day of surgery to treatment; Kuopio – Graphpad Quikcalcs (http://www.graphpad.com/quickcalcs/) was used to randomise animals to treatment; Lubeck – animals selected at random from cages and assigned either treatment A or B; Manchester – randomisation sequence generated in Microsoft Excel, and animals numbered in advance with selection in order of numbering on day of surgery. All assessments were blinded in accordance with ARRIVE^15^ and STAIR^19^ guidelines.

#### Exclusion criteria

Data were excluded from the study if the following were observed: (i) no sustained reduction (≥80% of baseline) in cerebral blood flow (CBF) during occlusion, (ii) lack of reperfusion (where relevant i.e. transient MCAO), (iii) any sign of subarachnoid haemorrhage/blood vessel rupture, (iv) respiratory distress/arrest during induction and/or maintenance of anaesthesia, (v) heart failure during surgery, (vi) seizure activity (short- or long-term), (vii) severe and prolonged weight-loss (typically > 15% over 48 h period). Exclusion of any data was done prior to unblinding of the study.

#### Ethics

Each participating lab conducted all *in vivo* procedures in accordance with the European Communities Council Directive (86/609/EEC) and local/national ethical rules and legislation as follows: Budapest – the Animal Care and Use Committee of the Institute of Experimental Medicine, Budapest, Hungary; Caen – in accordance with French ethical laws (act no. 87–848; Ministère de l’Agriculture et de la Forêt) and approved by the local ethical committee (authorisation code CENOMEXA 0113-03); Kuopio – the National Animal Experiment Board in Finland (ELLA) under license ESAVI-2011-000855; Lubeck – all animal experiments were approved by the local animal welfare committee (Ministerium für Energiewende, Landwirtschaft, Umwelt und ländliche Räume; Manchester – all procedures were performed under relevant personal and project licences and adhered to the Animals (Scientific Procedures) Act, UK (1986).

### Data extraction

Individual data were sought for each animal from each leading study investigator by a reviewer (RW) unaffiliated in conducting the studies. All included data were unpublished. Data retrieved included species, sex, age; outcomes including functional (e.g. corner test), neurological scores (e.g. Bederson), oedema (staining or MRI), lesion volume (MRI or staining); and vital status (including information on timing and cause of death – surgery, culling due to poor health, spontaneous). Information on treatment was also obtained: time of treatment from occlusion time (hours before/after), loading and maintenance dose of IL-1Ra.

The following study design information was extracted: experimental model – transient, permanent; randomisation – randomised, pseudo-randomised, not randomised; blinding of surgeon to treatment; blinding of outcome assessors to treatment. Studies were considered randomised if animals were numbered before commencement of the study, and a randomisation code was used to allocate animals to treatment groups; if animals were ‘picked at random’ from a cage, then these studies were considered pseudo-randomised since this type of approach is open to bias.

### Data analysis

Data were transferred to the project’s coordinating centre in Manchester by email attachment, e.g. Excel file. Study datasets were merged into a single Microsoft Excel sheet using common field names with one row per animal for analysis. Due to the heterogeneity in study design, no single outcome measure was assessed in the same way across the individual experiments. Therefore, based on the dataset available, it was decided to perform a meta-analysis of the individual studies, with lesion volume, oedema, neurological deficit, functional outcome and mortality as measures of outcome. Cochrane Review Manager (version 5.2) was used to analyse the effect of IL-1Ra treatment compared to vehicle on post-stroke outcomes. To normalise for differences in the absolute lesion volume obtained across individual centres, individual lesion volumes were recorded as cube root transformations. Oedema values were reported as either absolute volume or as a percentage of the intact contralateral hemisphere. IL-1Ra dose was standardised to mg/kg. In order to combine the different units, stroke outcomes were standardised by standard mean difference (SMD), i.e. the difference in means/standard deviation of score, except for lesion volume for which all values obtained were absolute and so outcome was reported as mean difference. An SMD/mean difference of zero represents a lack of intervention effect, while a positive or negative value represents the intervention that favours one treatment compared to the other. For the corner test, it was unknown whether the trials in studies were up to a certain number of turns or timed, hence SMD was used for analysis. Post-treatment death was compared by odds ratio (OR) analysis. Statistical heterogeneity was accounted for through the use of the DerSimonian and Laird^20^ pooling model of random effects in all analysis except for functional test and mortality, where fixed effects were used. Meta-analyses of IL-1Ra treatment versus vehicle were carried out by types of outcome, including quantification of lesion volume or oedema by staining method or MRI, neurological scores, corner test and post-treatment deaths. Outcome data were stratified into days of assessment if outcomes were measured on multiple days. Publication bias was not assessed in this meta-analysis, since all available data were obtained from participating labs.

Data are given as SMD (continuous data), mean difference (continuous data) or ORs (binary data) with 95% confidence intervals (95% CI), *P* value for effect, *P* value for heterogeneity and *P* value for interaction; *P* values <0.05 are considered significant. Negative coefficients imply a reduction in lesion volume. An OR less than one implies a reduction in death.

## Results

### Dataset characteristics

The dataset characteristics of the effects of exogenously administered IL-1Ra versus vehicle on outcomes after cerebral ischaemia are reported in Table 1. Datasets were retrieved from eight individual studies conducted across five different labs and countries (three in the UK (single lab), one in France, one in Finland, one in Hungary and one in Germany). Two studies were split into two datasets each due to differences in occlusion method (filament or thrombin) and whether recombinant tissue plasminogen activator (rtPA) was added with treatment or not. Data from a total of 241 experimental animals were retrieved.

### Lesion volume

**Figure 1.**
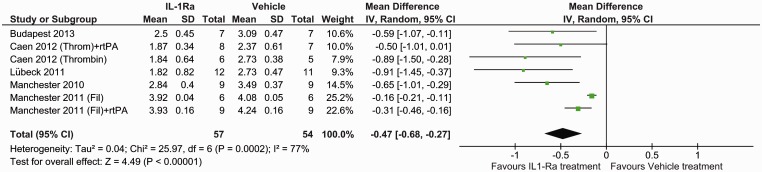
Forest plot of the effect of IL-1Ra on lesion volume measured by histology.

**Figure 2.**
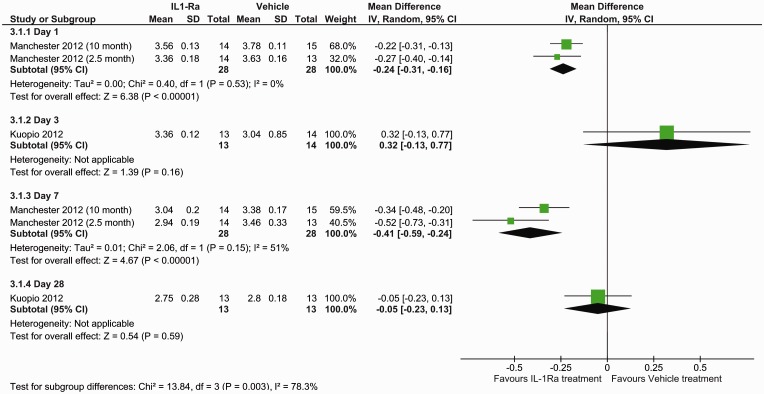
Forest plot of the effect of IL-1Ra on lesion volume measured by MRI.

The effect of IL-1Ra on lesion size was assessed using histological staining at seven days (five studies, seven datasets) and MRI at different times (three studies). With histological evaluation IL-1Ra reduced lesion volume, in comparison with vehicle: mean difference −0.47 (95% confidence interval (CI) −0.68 to −0.27, *P* ≤ 0.0001) (Figure 1). This corresponds to a mean reduction in lesion volume of 42.9% (95% CI 24.5–61.2%), as determined from the effect sizes across individual studies. Analysis of MRI determined lesion volumes also found a reduction following IL-1Ra treatment: mean difference −0.24 (95% CI −0.37 to −0.11, *P* = 0.0003) (Figure 2). Subgroup analysis of MRI lesion volumes found IL-1Ra treatment to reduce lesion size at days 1 (*P* ≤ 0.0001) and 7 (*P* ≤ 0.0001), but not at day 3 (*P* = 0.16) and day 28 post surgery (*P* = 0.59), though these data are derived from just two studies (one experimenter) at days 1 and 7, and a single study at days 3 and 28 (Figure 2).

### Oedema

**Figure 3.**
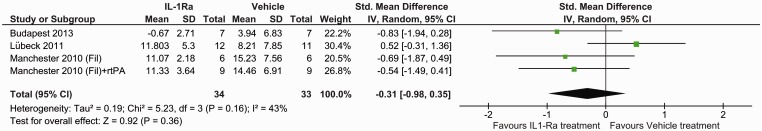
Forest plot of the effect of IL-1Ra on oedema measured by histology.

**Figure 4.**
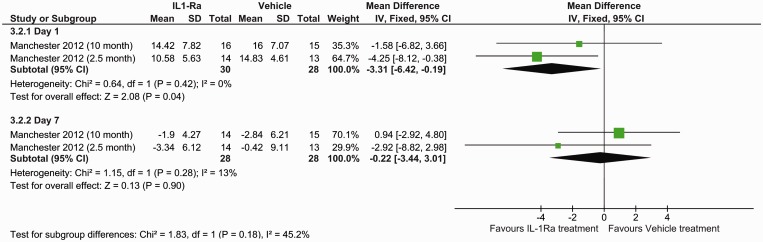
Forest plot of the effect of IL-1Ra on oedema measured MRI.

Oedema data were retrieved from three studies (four datasets), which obtained their oedema measurements via stained sections and two studies using MRI. Data were available at day 7 for both methods but only at day 1 for MRI. Histological staining (SMD: −0.31, 95% CI −0.98 to −0.35, *P* = 0.36) (Figure 3) and MRI measurements (*P* = 0.90) (Figure 4) found no benefit of IL-1Ra treatment in reducing oedema volume at day 7. However, oedema measured by MRI did show IL-1Ra treatment to be beneficial on day 1 (*P* = 0.04).

### Neurological deficit

**Figure 5.**
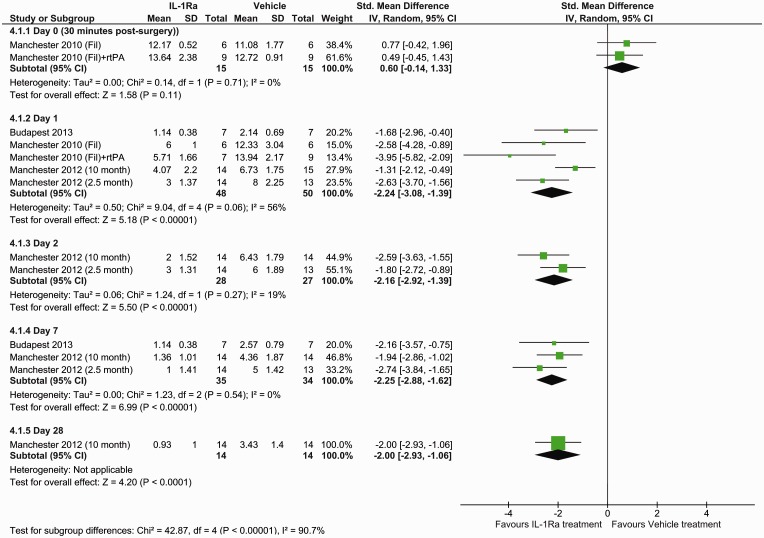
Forest plot of the effect of IL-1Ra on neurological deficit.

A total of four studies (five datasets) were available for neurological deficit score analysis (Figure 5). Neurological scores showed IL-1Ra treatment to reduce neurological at days 1 to 28 (*P* ≤ 0.001) but not shortly after surgery (*P* = 0.11).

### Functional outcome

**Figure 6.**
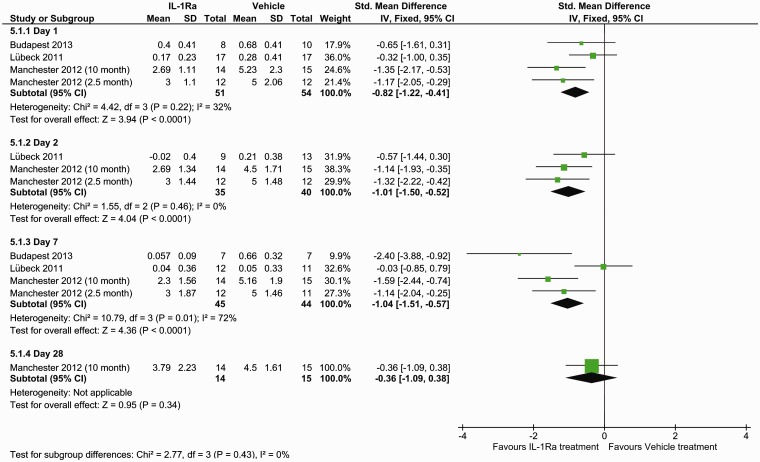
Forest plot of the effect of IL-1Ra on corner test.

Corner test data were available from four studies and overall analysis found IL-1Ra treatment to be beneficial at days 1 (*P* = 0.001), 2 (*P* ≤ 0.001) and 7 (*P* = 0.01) after stroke but not at day 28 (*P* = 0.34) (Figure 6). Gait was measured by the catwalk method in a single study only, so was not analysed in this meta-analysis.

### Mortality post treatment

**Figure 7.**
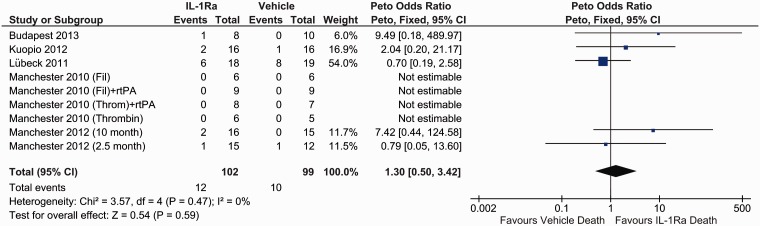
Forest plot of the effect of IL-1Ra on mortality post treatment.

Post-treatment mortality analysis revealed no significant difference between IL-1Ra and vehicle treatment (OR 1.30, 95% CI 0.50 to 3.42, *P* = 0.59) (Figure 7). There were a total of 22 deaths post surgery with 9 other animals excluded from analysis due to death during surgery (n = 5) or exclusion for other reasons (n = 4).

## Discussion

The objectives of this cross-laboratory study were to evaluate short-term and long-term effects of IL-1Ra on stroke outcome after MCAo in rodents, and to establish the feasibility and challenges faced in running a multicentre preclinical project. To our knowledge, this is one of the first cross-laboratory preclinical trials conducted. A meta-analysis of the data was necessary, since no one factor was measured uniformly across all animals, preventing a grouped analysis as originally planned. Despite this, the meta-analysis found IL-1Ra treatment to be beneficial in terms of reducing lesion damage, neurological deficit and improving functional outcomes after experimentally induced stroke in mice.

There has been a great disconnect between preclinical studies and human clinical trials. New therapies or interventions shown to be effective in animal studies of stroke have all failed when taken to clinical trial.^21^ Numerous reasons could explain this but in general preclinical studies are not always representative of human clinical trials, particularly with regard to the lack of multiple site assessment, with single site assessment of efficacy being the norm. Implementing cross-laboratory studies could therefore bring us one step closer to successful translation.

The use of different models was well represented in this cross-laboratory study with a mix of both transient and permanent models. Also, the majority of studies had data for both functional and lesion volume. A SOP was provided to participating centres; however, this was not typically followed, for a variety of reasons, not least local capabilities and regulations. Differences in animal housing surgical protocols and outcome assessments therefore increased the heterogeneity within the study. Where possible, we introduced measures to replicate some of these factors in at least one additional lab. For example, although the use of C57BL/6 mice was advised this proved not to be possible in one site, where BALB/C mice that were moderately aged (10 months) were used instead. We therefore replicated this study in another site to allow better comparison of the results.

In clinical trials, the choice of outcome measure can determine the success or failure of putative therapeutic intervention.^21^ In this cross-laboratory study, there was in the end a lack of defined endpoints and no single primary outcome that was assessed uniformly in all participating labs, despite providing an SOP. This was unexpected and any future multicentre studies should ensure that participating centres are able to follow closely the SOP, particularly with respect to outcome measures, identifying a primary outcome that is assessed the same in all studies. Harmonisation of procedures and the provision of SOPs that could be applied across all centres would also be beneficial. However, though we were not able to obtain data in all animals for a single outcome and ended up with a lot of heterogeneity, this could actually be considered a strength of the study. There has been much criticism that preclinical stroke studies lack the heterogeneity observed in human stroke and that the variation in our study is actually quite ‘translational’ and strengthens the data rather than reduces their reliability. In this study, the efficacy of IL-1Ra was demonstrated in different strains of mice with different occlusion methods and across several outcome measures.

In terms of lessons learnt from running this study, centralised collection, preparation and analysis of brains to determine lesion volume for example would be advantageous. Similarly, behaviour could be videoed locally and sent for analysis across several sites to ensure consistency. The study design should also be established clearly from the outset, with the total number of animals for the whole study being determined through appropriate power analysis. Participating centres would then receive instructions as to how many animals to include in the local study. Randomisation and allocation of treatment should also be coordinated centrally to ensure consistency and treatment vials should be individually numbered rather than just labelled A or B. It should be noted that this study was conducted very much as proof-of-principle and with very limited resources, participating labs kindly agreeing to perform the experiments within their own budgets. Clearly, any future multicentre preclinical trial would need to utilise more than one species, introduce aged animals and other co-morbidities, and assess effects in both male and females. A framework on which to run multicentre preclinical trials is currently being developed as part of an EU Framework seven funded project MultiPART (http://www.dcn.ed.ac.uk/multipart/), and the lessons learned from this study and others with cross-laboratory effort are being fed into this.

In conclusion, IL-1Ra treatment was beneficial overall in this cross-laboratory study in terms of reducing lesion damage, neurological deficit and improves functional outcomes after experimentally induced stroke in a specific subpopulation of young to middle-aged male mice. Furthermore, we show that cross-laboratory preclinical studies are feasible, but require careful planning and a clearly defined experimental design.
